# Comprehensive mapping of exon junction complex binding sites reveals universal EJC deposition in *Drosophila*

**DOI:** 10.1186/s12915-023-01749-1

**Published:** 2023-11-07

**Authors:** Lucía Morillo, Toni Paternina, Quentin Alasseur, Auguste Genovesio, Schraga Schwartz, Hervé Le Hir

**Affiliations:** 1 Institut de Biologie de l’École Normale Supérieure (IBENS), École Normale Supérieure, CNRS, INSERM, Université PSL, Paris, 75005 France; 2 Department of Molecular Genetics, Weizmann Institute of Science, Rehovot, 7630031 Israel

**Keywords:** Exon junction complex, eCLIP, RBPs, mRNP, RNA-protein interaction, Transcriptome-wide mapping

## Abstract

**Background:**

The exon junction complex (EJC) is involved in most steps of the mRNA life cycle, ranging from splicing to nonsense-mediated mRNA decay (NMD). It is assembled by the splicing machinery onto mRNA in a sequence-independent manner. A fundamental open question is whether the EJC is deposited onto all exon‒exon junctions or only on a subset of them. Several previous studies have made observations supportive of the latter, yet these have been limited by methodological constraints.

**Results:**

In this study, we sought to overcome these limitations via the integration of two different approaches for transcriptome-wide mapping of EJCs. Our results revealed that nearly all, if not all, internal exons consistently harbor an EJC in *Drosophila*, demonstrating that EJC presence is an inherent consequence of the splicing reaction. Furthermore, our study underscores the limitations of eCLIP methods in fully elucidating the landscape of RBP binding sites. Our findings highlight how highly specific (low false positive) methodologies can lead to erroneous interpretations due to partial sensitivity (high false negatives).

**Conclusions:**

This study contributes to our understanding of EJC deposition and its association with pre-mRNA splicing. The universal presence of EJC on internal exons underscores its significance in ensuring proper mRNA processing. Additionally, our observations highlight the need to consider both specificity and sensitivity in RBP mapping methodologies.

**Supplementary Information:**

The online version contains supplementary material available at 10.1186/s12915-023-01749-1.

## Background

The perception of cellular messenger RNAs (mRNAs) has evolved significantly over the last decade. Protein-coding transcripts are often perceived as long linear and mostly unstructured molecules. However, mRNAs, even those of several kilobases of length, are largely covered by RNA-binding proteins (RBPs) to form large and compact messenger RiboNucleoProtein (mRNP) particles [1]. Upon translation, these compact mRNP structures undergo a process of decompaction [2, 3]. RBPs are essential to dictate the complex life of mRNAs from nuclear processing to cytoplasmic translation into proteins and ultimately degradation [4].

The exon junction complex (EJC) is an abundant component of mRNPs that is loaded during the splicing reaction by the spliceosome onto the mRNA upstream of exon‒exon junctions [5–7]. The core of the EJC consists of three proteins, eIF4A3/DDX48, MAGOH/MAGO, and Y14/RBM8A. eIF4A3 is a DEAD-box helicase that binds to the sugar–phosphate backbone of mRNA independently of the sequence, while the heterodimer MAGOH-Y14 locks eIF4A3 onto the RNA [8–10]. Once clamped on mRNA, the EJC acts as a binding platform for several peripheral factors both in the nucleus and, after mRNA export, in the cytoplasm. Finally, EJCs are disassembled by scanning ribosomes during the first round of translation, even if a translation-independent EJC disassembly is not excluded [11]. The EJC participates in several steps of mRNA processing, including pre-mRNA splicing, nuclear export, RNA localization, translation, and decay [6, 7]. Recent studies have unveiled an additional function of the EJC in shaping the distribution of the RNA modification N6-methyladenosine (m^6^A) [12] by excluding m^6^A around spliced junctions [13–15]. This function could be caused by the role of EJC in the 3D organization of mRNPs [14]. EJCs in multiple copies along spliced transcripts could interact with each other and other RBPs, making them cornerstones of mRNP architecture [16–18]. Taken together, the EJC is crucial for both mRNP structure and for the successive steps of mRNA existence.

The EJC was initially assumed to be present on every exon‒exon junction based on its loading mode, which is splicing-dependent and sequence-independent [19]. However, this notion has been challenged by both low-throughput methods, such as reporter expression [20, 21] and immunoprecipitation (IP) [22], and high-throughput methods involving various RNA isolation protocols coupled to sequencing [16, 23–27]. Although these studies established that EJCs are deposited on average approximately 25 nucleotides upstream of spliced junctions, they failed to detect an EJC present on all junctions. In humans, approximately 80% of exons were found to contain an EJC in the canonical region (-24 nt) [16, 23]. In contrast, studies in *Drosophila* did not provide transcriptome-wide information at the exon level, but only 42% of genes were found to carry an EJC [27], and reporter expression in *Drosophila* embryo showed that not all exons are associated with an EJC [20].

Mapping the interaction sites of RBPs on mRNA poses specific challenges and complexities [28, 29]. It requires isolating the RBP of interest from other RBPs while preserving its specific interaction with the mRNA. Several techniques have been developed for this purpose, the main ones being RNA IP followed by sequencing (RIP-seq) and RNA IP sequencing after RNA‒protein crosslinking by UV (CLIP-seq [30]) or after protein-protein crosslinking [16, 27]. RIP-seq analyzes native mRNA under low stringency conditions to preserve RNA‒protein interactions, but this can result in the co-IP of multiple proteins and their associated RNA molecules. In contrast, UV cross-linking methods, such as CLIP, emerged as a significant breakthrough in the field since cross-linked RNA-proteins sustain stringent RNA isolation procedures that avoid contaminating RBPs. Methodological advancements have facilitated the identification of binding sites at nucleotide resolution [29, 30]. However, this approach comes with major drawbacks, notably the low crosslinking efficiency, which can result in a low signal-to-noise ratio causing limited sensitivity, and a crosslinking bias toward certain nucleotides [28].

In this study, we conducted enhanced CLIP sequencing (eCLIP [31]) to identify eIF4A3 binding sites in both human and *Drosophila* transcriptomes. Additionally, we incorporated a new analysis from a previously published eIF4A3 interactome in *Drosophila* [27]. By focusing on *Drosophila*, utilizing triplicates, and combining different techniques, we successfully overcame the challenging low reproducibility issues common to eCLIP studies. Our findings provide compelling evidence that the current understanding of the EJC interactome is highly influenced by technical limitations. Tackling those limitations allowed us to infer that nearly 100% of exon junctions are loaded with EJCs.

## Results

### Transcriptome-wide eIF4A3 eCLIP-seq peaks show poor reproducibility in human cells

**Fig. 1:**
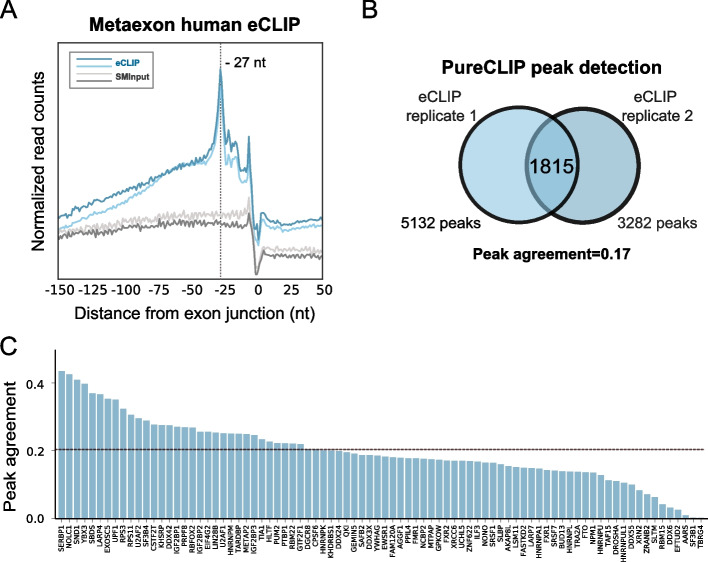
Reproducibility of transcriptome-wide eIF4A3 eCLIP-seq peaks in human cells. **a** The positioning of 5′ ends of human eIF4A3 eCLIP reads relative to the exon junction for two eCLIP replicates (in blue) and two SMInputs replicates (in gray). The truncation signal is normalized by both exon distribution and library size. The eCLIP signal peaks at a position 27 nucleotides (nt) upstream of exon-exon junctions. **b** A Venn diagram illustrating eIF4A3 peaks detected by PureCLIP in eCLIP replicates 1 and 2. The number of peaks in common or not between the two replicates is indicated. The peak agreement between the two replicates corresponds to the calculated Jaccard index (JI). **c** A barplot showing the distribution of Jaccard index values for peaks detected using PureCLIP analysis of replicates of eCLIP datasets for 84 different RBPs from ENCODE. The median value of the distribution is 0.2 (dotted line)

To comprehensively analyze the EJC binding profile across the transcriptome in human cells, we conducted eIF4A3 eCLIP sequencing using a HeLa cell line in which the endogenous eIF4A3 protein is fused to 3x HA tags to improve both the specificity and efficiency of IP [26]. We produced and sequenced two eIF4A3 eCLIP replicates (eCLIP1 and eCLIP2) as well as two size-matched inputs (SMInputs 1 and 2) controls to identify the most abundant nonspecific RNA fragments contributing to the background signal [31] (Additional file 1: Fig. S1a). eCLIP signal interpretation relies on read truncations corresponding to reverse transcriptase (RT) arrests at crosslinking sites. To observe the EJC binding profile, we computed the distance of the 5′ end of each read to the corresponding end of the exon (Fig. 1a). A meta-exon plot confirmed a signal enrichment at 27 nucleotides upstream of the exon junction, verifying the expected EJC binding site detected by eCLIP [26]. The large spread of 5′ ends upstream of the main peak is attributed to readthrough events resulting from the RT bypassing the crosslinking site [26].

A widely employed method for eCLIP analysis is PureCLIP, which combines eCLIP-specific truncation patterns and mRNA-enriched regions to identify individual crosslink sites [32]. With PureCLIP approximately 7000 and 5000 peaks were identified for the eCLIP1 and eCLIP2 samples, respectively (Fig. 1b, Additional file 2: Table S1). Binding site reproducibility is a fundamental metric for assessing the accuracy of a method, as it ensures confidence in the identified binding sites and reduces false positives arising from high background noise [33]. Therefore, to measure reproducibility between the peaks detected in both replicates, we use the Jaccard index, which is the size of the intersection between two datasets divided by the size of their union, indicated in the figure as *Peak agreement*. In human eIF4A3 eCLIP, the Jaccard index was only 0.17 (Fig. 1b), indicative of poor experimental reproducibility (Additional file 2: Table S1).

To test if this poor reproducibility was eIF4A3 specific, we determined the Jaccard index of 84 different human RBPs for which PureCLIP analyses of eCLIP data are publicly available [31]. The Jaccard indexes vary from almost zero to 0.42 with a median value of 0.20 (Fig. 1c). The Jaccard index obtained for eIF4A3 is thus well within the range of values across previous eCLIP datasets, suggesting that low reproducibility is a general feature in eCLIP datasets.

### eIF4A3 eCLIP-seq in *Drosophila* S2 cells shows higher peak reproducibility

The sharp peak in the human data, precisely at the anticipated position, suggested that the specificity of our data was high. We therefore hypothesized that the low reproducibility across replicates may not have been a consequence of poor specificity (and hence an abundance of false positives) but instead of poor sensitivity (and hence an abundance of false negatives). Given that UV crosslinking is highly inefficient and the human transcriptome is highly complex, this can lead to stochastic detection of sites, manifesting in low overlaps across replicates. To overcome this problem and ensure maximal sensitivity of detection, we therefore decided to modify our approach in two important ways: (i) to monitor EJCs within a species with substantially lower transcriptome complexity and (ii) to obtain deeper sequence coverage. Accordingly, we performed eIF4A3 eCLIP experiments in *Drosophila* melanogaster (*Drosophila*). Compared to the human transcriptome, the *Drosophila* transcriptome is simpler. There is a lower number of coding genes, approximately 13,000 in *Drosophila* versus 25,000 in humans [34]. More significantly, the *Drosophila* genes have a simpler gene architecture with fewer exons and introns (Additional file 1: Fig. S2a and S2b). In addition, *Drosophila* genes are subjected to fewer alternative splicing events than human genes, thereby sharply reducing the total number of exon‒exon junctions [26].

We followed the same experimental strategy as in HeLa cells by fusing a 3xHA tag to the endogenous eIF4A3 expressed in S2R+ *Drosophila* culture cells. The tagged protein is expressed at a level similar to the untagged protein in the wild-type cell (Additional file 1: Fig. S2c). We performed three eIF4A3 eCLIP replicates and two SMInputs. We acquired 2–10-fold more usable reads from these cells than from human cells (Additional file 1: Fig. S1b). Considering that the *Drosophila* transcriptome is less than half the size of the human transcriptome, the coverage of *Drosophila* eCLIP is significantly higher than that of human cells.

**Fig. 2:**
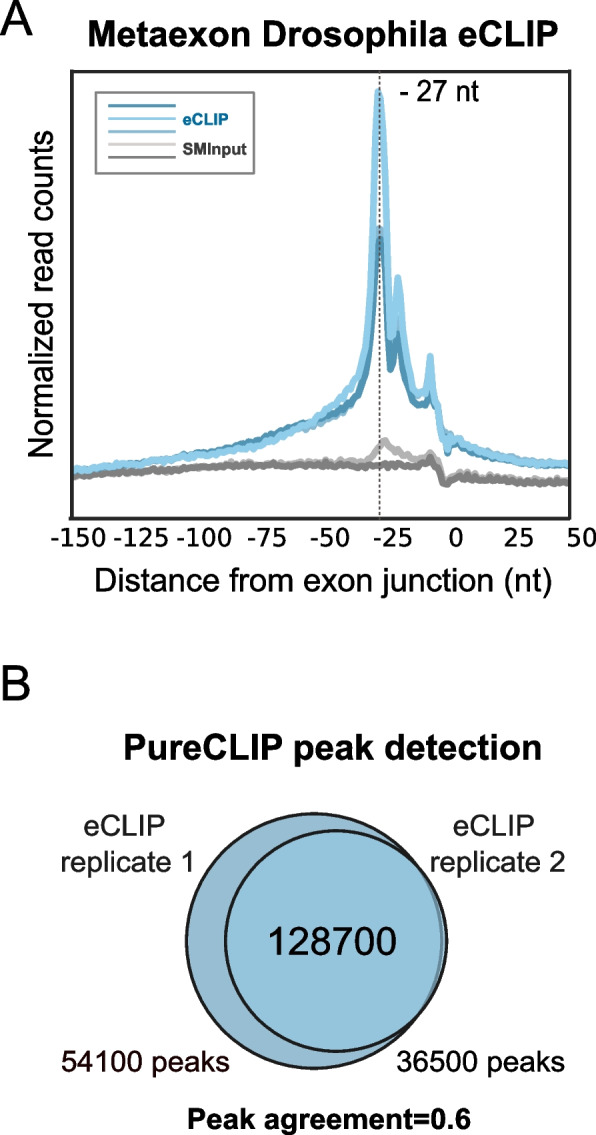
Reproducibility of transcriptome-wide eIF4A3 eCLIP-seq peaks in *Drosophila* cells. **a** The positioning of 5′ ends of eCLIP reads relative to the exon junction for three eCLIP replicates (in blue) and two SMInputs replicates (in gray). The truncation signal is normalized by both exon distribution and library size. The eCLIP signal peaks at a position 27 nucleotides (nt) upstream of exon-exon junctions. **b** A Venn diagram illustrating eIF4A3 peaks detected by PureCLIP in eCLIP replicates 1 and 2. The number of peaks in common or not between the two replicates is indicated. The peak agreement between the two replicates corresponds to the calculated Jaccard index (JI)

A meta-exon plot of the 5′ extremity of *Drosophila* eIF4A3 eCLIP reads shows a major accumulation of crosslinking sites 27 nts upstream of exon junctions, as in the human eIF4A3 eCLIP (Fig. 2a). Therefore, EJC positioning by the splicing machinery is well conserved between the two organisms. In *Drosophila*, the larger peak-to-background ratio indicates lower noise levels compared to human data (Figs. 1a and 2a). Moreover, the sharper profile observed in the − 27 peaks, with a decrease in upstream signal compared to the human meta-exon, can be attributed to two factors. First, it may be a consequence of fewer read-through events occurring in *Drosophila*. Second, it could be due to a more accurate mapping of eCLIP reads onto the *Drosophila* transcriptome due to its simplicity, resulting in a more precise alignment of the binding sites. These observations indicate that the increased coverage achieved in *Drosophila* resulted in a significant increase in the signal-to-noise ratio. PureCLIP analyses of *Drosophila* eCLIP datasets yielded between 50 and 150 thousand called peaks (Fig. 2b, Additional file 1: Fig. S1c). Remarkably, the different replicates gave Jaccard index values between 0.6 and 0.7, much higher than in human (Fig. 2b, Additional file 1: Fig. S1c), indicative of a significantly higher sensitivity and reproducibility of eIF4A3 eCLIP peaks in *Drosophila* compared to human cells.

### Stop rate difference, a reproducible metric for eCLIP peak detection

**Fig. 3:**
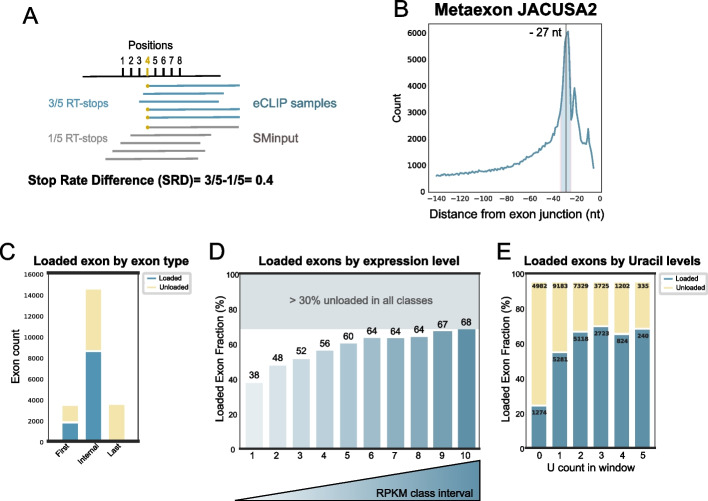
eCLIP data analysis with JACUSA2. **a** Schematic representation of the JACUSA2 principle. Fragments from eCLIP and SMinput datasets generated by Reverse transcription (RT) stop at different positions indicated on the top. As an example, the Stop Rate Difference (SRD) calculation is indicated for the position 4 (yellow dots). **b** Distribution of positions with Stop Rates that have a *p*-value < 0.05, plotted relative to the exon junction. The vertical gray line represents the position − 27, while the blue area that spans from − 23 to − 32, corresponds to the window used for calculating the SRD score per exon. **c** Stacked bar plot showing the count of detected exons and unloaded exons per exon group (first, internal, and last). **d** The bar plot illustrates the distribution of detected exons in different expression classes for exons from all genes, excluding the last exon. Each bar represents a bin of equal size, and the percentage of loaded exons out of the total is indicated on top of each bar. **e** Stacked bar plot correlating uridine count in the previously selected window with the relative percentage of exons corresponding to either detected or undetected categories. The numbers indicated in each bar represent the total number of exons (top) and the number of loaded exons (below)

Based on the above results, we decided to focus our efforts on *Drosophila*. To move from quantifications at the metagene level to the detection of individual cross-linked sites and exons, we implemented an approach relying on the detection of RT-arrests induced by cross-linking (‘stop rate scores’). RT-arrests were quantified via JACUSA2 software. JACUSA2 rt-arrest was originally employed to map RNA modifications [35]. This method is centered on calculating RT-arrests per position and normalizing them by coverage per position. Then the stop rate score per position was further normalized by the stop rate score of the input, giving the stop rate difference (SRD) (see the “Methods” section) (Fig. 3a). Posteriorly, an additional exon-centered score was established by calculating the average SRD score of all significant positions within a 10-bp window around the 27th nucleotide upstream of the exon junction, giving an exon-level SRD score. Several lines of analysis confirmed the adequacy of this approach: (i) Sites with significant SRD scores per position were narrowly distributed around position − 27, as expected (Fig. 3b). As previously observed on the basis of the metagene analysis (Fig. 2a), a secondary peak at − 18 is reproducible even when considering only statistically significant positions. Posteriorly, an *rt-arrest* study focusing on a window around position 18 (see the “Methods” section) showed that 70% of peaks at − 18 coexisted on the same exons that harbored a major peak. However, its origin will require future characterization. (ii) We observed a higher reproducibility between eCLIP replicates when comparing Stop Rate Scores (Additional file 1: Fig. S3a) than with PureCLIP scores (Additional file 1: Fig. S3b). This increase in similarity between replicates was not accompanied by a loss in the number of detected sites, which remained similar (~20 thousand peaks for PureCLIP replicates and ~15 thousand peaks for SRD, commonly detected in all replicates) (Additional file 2: Table S2). (iii) EJC deposition is not expected to occur on the last exon, which lacks a downstream intron and hence is not subjected to splicing. Therefore, the presence of a signal on the last exon is likely a false positive. We classified exons with a positive SRD into the three following classes: first exon, internal exons, and last exons. The almost complete depletion of the signal in the last exon (0.4%) argues for the specificity of detection by SRD (Fig. 3c, Additional file 2: Table S3).

We next analyzed the “EJC loaded” exons, which correspond to exons associated with a positive SRD score per exon. Considering all *Drosophila* internal exons, we only detected a significant stop rate score of 59% (21% for first exons) (Additional file 2: Table S3). However, careful examination of the data revealed two strong sources of biases, both of which led to a severe underestimation of this fraction. First, detection is clearly biased by expression levels. To evaluate this bias, the expression of each exon was estimated by mRNA-seq, and all exons were classified into 10 equal classes of increasing expression bins. The percentage of loaded internal exons was approximately 40% for the lowest expressed ones and reached ~70% for the most highly expressed ones (Fig. 3d). Second, we observed a bias related to the Uridine (U) content of exons. All CLIP-related methods are biased at some level by the nucleotide composition of the RBP binding site due to crosslinking preferences, notably for uridines [36]. To evaluate the contribution of U content to EJC detection, we selected a window of 7 nucleotides around position − 27, the selection of the window was based on the structural knowledge that the EJC covers 8 nucleotides [8]. Then, we counted the number of Us within the region for each exon and correlated that with EJC loading. In the absence of U within the crosslinking region, only 25% of exons were detected with an EJC, whereas the presence of just one U increased detection levels to 60% (Fig. 3e). Importantly, this Uracil bias held consistently across all expression levels. In instances where U was absent in the crosslinking regions, irrespective of the expression category, it resulted in low SRD scores. Conversely, an increase in U counts directly correlated with higher SRD scores (Additional file 1: Fig. S3c). Consequently, the proportion of eIF4A3-loaded exons detected by eCLIP is largely underestimated.

Our analyses highlight the suitability of the SRD metric for eCLIP peak detection. Moreover, by acknowledging and accounting for these technical biases, we can improve the interpretation of eCLIP results, leading to more robust and accurate insights into RNA‒protein interactions.

### Combination of eCLIP and ipaRt data analysis establishes universal EJC loading on exon junctions

**Fig. 4:**
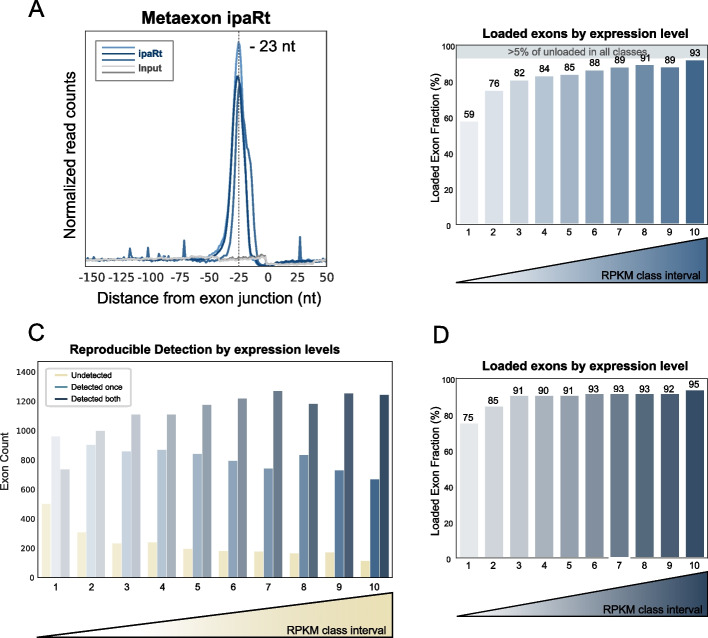
Combination of eCLIP and ipaRt data analysis establishes the universal EJC loading. **a** Distribution of the middle position of ipaRt reads relative to the exon junction for three replicates (in blue) and two control mRNA-seq (in gray). The signal peaks at a position 23 nucleotides (nt) upstream of exon-exon junctions. **b** A bar plot illustrating detected exons by ipaRt in 10 different expression classes (RPKM class interval). Classes were created with equal bin sizes and for exons from all genes excluding the last exons. The percentage of loaded exons is indicated on top. **c** A bar plot showing, among all commonly detected genes, the number of exons detected as loaded in both experiments (in dark blue), unloaded in both experiments (in yellow), and variably detected between conditions (in light blue) within each expression class. **d** A bar plot representing detected exons in at least one of the datasets in different expression classes with equal bin sizes for exons from all genes excluding the last exon. The percentage of loaded exons is indicated on top

Given the observed constraints of eCLIP, we complemented our EJC mapping by re-analyzing a previous transcriptome-wide EJC interactome established in *Drosophila* by an RNA IP strategy independent of UV crosslinking [27]. In this study, the authors developed the method ipaRt (isolation of protein complexes and associated RNA targets) in which *Drosophila* extracts are treated with a protein‒protein crosslinking agent to stabilize EJCs onto RNA before isolation of EJC-bound RNA fragments. In comparison to eCLIP, examination of IGV coverage revealed a very clear signal enrichment upstream of exon junctions for the ipaRt method, characterized by lower background noise (Additional file 1: Fig. S4a). While ipaRt does not, inherently, provide data at single nucleotide resolution (in contrast to eCLIP), we found that when we considered the middle of each read as a proxy for the whereabouts of the EJC, we observed a sharp peak 23 nt upstream of the exon junction (Fig. 4a). The slight deviation from the − 27-position observed with eCLIP data (Figs. 1a and 2a) is most likely because the position of the middle of EJC-bound reads purified by ipaRt does not exactly correspond to the crosslinked nucleotide mapped by eCLIP analysis. Nevertheless, the ipaRt dataset consistently confirms the highly conserved and precise binding site of the EJC onto the mRNA.

To enable exon-level binding analysis for ipaRt, we created a score based on an enrichment ratio between the median coverage per base in the EJC binding site window of ipaRt compared to the same window in the mRNA-seq (see the “Methods” section). To calibrate the threshold based on which exon was considered to harbor an EJC, we utilized the fact that the signal was by large absent in the last exon and hence considered the 95th percentile of signal in the last exon as a minimal threshold for considering an exon as loaded (Additional file 2: Table S4). We considered exons as loaded if they were detected in the 3 replicates. Considering all internal and first exons (commonly expressed between S2 cells and whole fly, for comparison purposes), we found that 84% were loaded. However, as in the eCLIP analysis, this analysis was strongly biased by expression levels, with only 60% of lowly expressed exons harboring an EJC but 93% of exons within highly expressed genes (Fig. 4b). In contrast to eCLIP, the fraction of detected exons was not biased by U count, since ipaRt does not rely on UV crosslinking (Additional file 1: Fig. S4c). Hence, this analysis suggests that the vast majority of internal exons are associated with an EJC.

The ipaRt method offers advantages in minimizing UV-induced biases and providing a more direct assessment of EJC binding, but a drawback of this method is that it results in the bridging of protein‒protein interactions in a global manner and consequently freezes complexes in a state that may not reflect their dynamics. This disadvantage is not shared by the eCLIP approach. To further improve the robustness of our analysis, we integrated the results from both methods. To do so, we first selected genes that were expressed and detected in both datasets. We next classified the exons from these genes into three categories: those detected by both methods (11,318), those detected by only one method (2377), and those not detected by either method (2377). Exons detected in only one method were mainly from ipaRt since, as previously mentioned, this method has a higher sensitivity than eCLIP. The reproducibly unloaded exons, as anticipated, were primarily associated with lower expression levels (Fig. 4c). Subsequently, we performed a combined analysis of both methods, considering all exons detected by either one or both approaches. This integration yielded a substantial increase in the number of detected exons across all classes. Remarkably, even the low expressed exons showed a significant improvement in detection, with approximately 90% of these exons being identified. This plateau in detection efficiency was achieved at a relatively modest expression level, suggesting that our combined approach effectively captures a comprehensive set of exons across the transcriptome. (Fig. 4d).

To ensure the undetected status of these exons, we selected the top 50 most expressed and reproducible undetected exons and visually inspected their binding profiles and associated RNA-seq data using the Integrated Genomics Viewer (IGV) (see Supplementary Material). However, we found it very challenging to find a single example of an internal gene failing to show evidence of an EJC. Specifically, the vast majority of the inspected sites fell into one of the following three categories: (i) Unexpressed exons in our cell line due to alternative splicing, (ii) exons that may be loaded but that did not pass our statistical thresholds (these may reflect false negatives), and (iii) Premature Termination Codon (PTC)-containing exons, which make transcripts rapidly targeted for degradation by the cellular machinery, making them impossible to detect in classic RNA-seq (Additional file 3: Table S5).

Previous studies showed that first exons were systematically less loaded with an EJC than internal exons [16, 27]. Interestingly, a notable proportion of the misclassified exons were found to be first exons. This observation can be attributed to the challenging task of characterizing these exons accurately, due to the extensive alternative promoter usage in *Drosophila* [34]. As a result, it appears that the loading of EJCs from first exons is as prevalent as that from internal exons.

In conclusion, despite the specific biases of both methods and that both studies were carried out using different starting cellular sources (extracts of adult *Drosophila* for ipaRt and S2 cells for eCLIP), by combining both methods, we observed that the majority of exons are reproducibly associated with an EJC and that those that did not mainly result from a very low expression level or a mischaracterization. Taken together, EJC deposition occurs on the vast majority, if not all, of exons, with the exception of the last exon of each transcript.

## Discussion

Different methods have been employed for isolating EJC-bound RNA fragments in order to obtain transcriptome-wide maps of EJC binding sites [16, 23–27]. All these studies, with more of less precision, consistently demonstrated a significant accumulation of EJC binding sites around 25 nucleotides upstream spliced junctions. The limited depth of most studies, mainly due to the inefficient UV crosslinking in eCLIP-seq experiments, and the lack of consideration of inter-replicate reproducibility at the exon level, limited our view of EJC binding sites to a meta-exonic perspective. However, these studies suggested a differential and regulated loading of the EJC across exon-exon junctions. The lack of EJC detection on certain junctions naturally led the authors to attempt to correlate the EJC presence with certain mRNA features including intron and exon sizes, splice site strength, alternative splicing events, or sequence motifs. However, all these efforts failed to establish an intelligible code that could explain a variable loading of the EJC. Each transcriptomic mapping strategy has its own limitations, which arise from the molecular tools used to purify mRNA fragments, from intricate mRNP particles in cellular extracts, as well as from the bioinformatic tools employed to analyze these sequenced mRNA fragments. Although optimization of these methods can enhance specificity, it often comes at the cost of reduced sensitivity. The issue of high specificity but low sensitivity is not exclusive to EJC mapping or CLIP sequencing. In the context of m6A detection, for example, it was previously shown that different methodologies — all with high specificity — can culminate in low overlaps of detected sites, due to low sensitivity of each of the methods, leading to high numbers of false negatives [14, 37]. By integrating datasets obtained through different experimental methods, it becomes possible to overcome individual limitations and improve specificity, leading to a more comprehensive understanding of the targeted molecular features [37]. In our study, we applied this strategy to map EJC binding sites into the *Drosophila* transcriptome. First, we isolated EJC-bound RNA fragments by performing eIF4A3 eCLIP. To improve the accuracy of EJC binding site discovery, we leveraged the high frequency of 5′ truncations relative to readthrough events by using JACUSA2 that prioritizes RT arrests over local enrichments. Depending on the RBP studied and its mode of RNA binding, it is important to adapt a proper peak caller pipeline to analyze CLIP data [38]. Additionally, we re-analyzed the available EJC interactome datasets obtained by RIP-seq after protein-protein crosslinking. These datasets were obtained independently of RNA-protein UV crosslinking, thereby avoiding the limitations and drawbacks associated with it [27]. By integrating both analyzes, we substantially increased the robustness of the results. Ultimately, we made the remarkable discovery that nearly every spliced junction of significantly expressed transcripts is associated with an EJC. Therefore, EJC deposition is a universal mark of pre-mRNA splicing.

The deposition of the EJC onto every mRNA spliced junction certainly constitutes an important attribute of spliced mRNAs. eCLIP-seq of eIF4A3 both in human [26] and in *Drosophila* (this study) gives an extremely sharp enrichment of EJC crosslinking sites at meta-exonic level, peaking 27 nucleotides upstream of spliced junctions in both cell types. This evolutionary conserved, spatially restricted and splicing-dependent loading of the EJC makes the existence of non-canonical EJCs not very likely and probably attributable to non-reproducible false-positive EJC peaks originally detected [16, 23].

In metazoans, most pre-mRNAs contain multiple introns [34]. Once loaded, the EJC hides and/or represses neighboring cryptic splice sites [39, 40]. Therefore, a universal deposition of EJCs would contribute to safeguard the integrity of the successive splicing events necessary to generate full-length and mature mRNAs [40]. In addition, given that EJCs constitute stable cornerstones for mRNP 3D organization [17, 18], the presence of an EJC on each spliced junction would ensure a homogeneous compaction of mRNP particles. This facet of EJC-dependent mRNP compaction is illuminated by the recent discovery that the presence of regularly spaced EJC prevents the deposition of m6A methylation onto mRNA leading to mRNA destabilization [13–15].

Several aspects of EJC existence remain to be solved or clarified. (i) Today, it remains speculative whether EJC deposition by the splicing machinery is also universal in other metazoans, notably in human. Here, we have taken advantage of the simplicity of the *Drosophila* transcriptome in terms of splicing events complexity compared to human. In the future, to extrapolate our conclusions and robustly map EJCs in humans, it will be crucial to enhance the sensitivity of methods used to detect EJC-bound targets. (ii) Our findings offer a *qualitative* view on EJC deposition, but fail to provide a *quantitative* view. Our findings establish that the vast majority of exon junctions have some extent or another of EJCs, but cannot address the EJC occupancy per exon, i.e., considering all transcripts harboring a certain junction, what fraction of these harbor an EJC. The lack of such precise measurements limits the ability to assess questions pertaining to EJC assembly, such as determining the per-exon EJC deposition rate. Moreover, this also renders it challenging to assess questions pertaining to the uniformity — or heterogeneity of EJC disassembly. While the prevailing model of the EJC suggests that it is deposited on every spliced junction and it remains stably bound until mRNA translation, EJC disassembly partially occurs in a translation-independent manner [11], rendering it interesting to dissect whether the residence time of EJCs vary from one junction to another and/or between transcripts, resulting in a variable amount of EJC between splice junctions. (iii) Moreover, individual spliced junctions have been shown to confer different functional output in an EJC-dependent manner both in *Drosophila* and in humans [22, 39–45]. A transcriptome-wide view of the differential EJC composition per exon will constitute an important step to understand EJC contribution to post-transcriptional gene regulation.

## Conclusions

In summary, our study provides important insights into the deposition of EJCs in pre-mRNA splicing. By employing a combination of transcriptomic mapping strategies, including eIF4A3 eCLIP and re-analysis of available EJC interactome datasets, we have clarified EJC deposition onto the *Drosophila* transcriptome. While the limitations of current methods hinder the ability to quantitatively assess EJC occupancy rates per exon and the dynamic nature of EJC disassembly, our study shows that EJC deposition is a universal mark of pre-mRNA splicing. We also showed that EJC deposition by the spliceosome is a conserved mechanism leading to a very precise positioning of the EJC upstream spliced junctions. Being a repressor of cryptic splice sites and a cornerstone of mRNP 3D organization, a systematic deposition of EJCs along transcripts would ensure the integrity of multi-intron-containing transcripts and a homogeneous compaction of mRNP particles. The sensitivity of EJC mapping must be further improved to extend it to other metazoan transcriptomes including the human one. Moreover, quantitative methods will be necessary to assess whether EJC residence time varies between exon-exon junctions and transcripts to fully comprehend the contribution of EJCs to post-transcriptional gene regulation.

## Material and methods

### Cell culture

Human HeLa cells were grown in Dulbecco’s modified Eagle’s medium (Gibco™) supplemented with 10% fetal bovine serum (PAN^TM^ BIOTECH), 100 U/mL penicillin/streptomycin (Life Technologies). Cells were passaged every 3–4 days and cultivated in a humidified incubator at 37 °C with 5% CO_2_.

S2 *Drosophila melanogaster* (*Drosophila*) cells were obtained from Arnaud Echard (Institut Pasteur) and cultivated in Schneider *D. melanogaster* Medium (Gibco™) supplemented with 10% fetal bovine serum (Life Technologies) and 1% penicillin/streptomycin (Life Technologies). Cells were maintained in a humidified incubator at 28 °C and split at 90% confluency using a 1/5 dilution, without trypsin reagent or PBS wash.

### Plasmids and molecular cloning for eIF4A3 genome editing

Genome editing of endogenous eIF4A3 in HeLa cells was accomplished following the methodology previously described [26]. For the endogenous tagging of S2 cells, we employed the Cas9 nickase from *Streptococcus pyogenes*, expressed using the pX335 plasmid (Addgene) along with sgRNAs derived from sgRNA expression vectors (kind gift from Edouard Bertrand [46]). SgRNAs targeting the C-terminal region of eIF4A3 were generated using Benchling software, synthesized by Eurofins, and cloned into the expression vectors using the golden gate assembly method. To create eIF4A3 homology regions, which consisted of 500 base pairs upstream and downstream of the stop codon and a modified PAM, gBlocks from IDT were amplified by PCR and cloned into vectors carrying the TEV-3xHA affinity tag, an internal ribosome entry site (IRES), a puromycin resistance gene, and the SV40 polyadenylation signal by Gibson assembly. Plasmid transfection was carried out using Lipofectamine (Life Technologies), with a 1:3 ratio of sgRNA plasmid to repair plasmid, following the manufacturer's protocol. After 24 h, cells were subjected to selection using puromycin at varying concentrations (0, 1, or 5 μg/ml). The selection process was conducted for 48 h, followed by single-cell dilution in a 96-well plate. Cells were kept on conditioned media for 1 month. Individual clones were then analyzed by PCR genotyping.

The sequences targeted by the gRNAs were:

- 5′AAACCGTTCATGGGCATCTCGTCGC 3′ Reverse
- 5′ TTCGCGACGAGATGCCCATGAACG 3′ Forward

The same procedure was followed to edit endogenous Y14 and add a FLAG tag. The sequences targeted by the gRNAs were:

- 5′ AACAGCCCCAAGAATAATTTTTTC 3′ Reverse
- 5′ TTCGAAAAAATTATTCTTGGGGC 3′ Forward

### Validation of eIF4A3-HA expression in *Drosophila*

Validation of the *Drosophila* eIF4A3-HA expression level was performed by Western blotting (WB) with both HA and eIF4A3 antibodies (Gift of Marco Blanchette, Stowers Institute, MO, USA) (Fig. S2b). Since EJC *Drosophila* antibodies are not commercially available, we used a S2 cell line carrying both edited eIF4A3-HA and Y14-FLAG, allowing us to perform co-IP of eIF4A3-HA and Y14-FLAG, using anti-HA (Sigma H6908) and anti-FLAG antibodies (Sigma F3165), validating EJC assembly (Fig. S2c).

### Oligonucleotides for eCLIP

Oligonucleotide design and RNA and DNA linker sequences from the published eCLIP procedure were modified to allow sequencing of the library in single-end mode and to be compatible with the P3/P5 PCR primers from Solexa. Random and multiplex barcodes were placed on the second ligation primer. Detailed sequences can be found in supplementary data from Hocq et al [26].

### eCLIP protocol

15 cm plate of S2 cells at approximately 80% confluency were UVC crosslinked (254 nm) at 150 mJ/cm2 followed by partial RNase I (Ambion) digestion. The soluble fraction was immunoprecipitated with an optimized volume of HA magnetic beads (70 µl; (Thermo Scientific)). Two percent of RNase-treated lysate was kept at 4°C for SMInput negative control. RNP complexes were washed stringently with a buffer containing 1 M NaCl and 2 M urea before cross-linked RNAs were 5′ and 3′ dephosphorylated, followed by 3′ RNA linker ligation (RT primers). The resulting ligated RNPs and SM-input control were purified by SDS‒PAGE and transferred onto a nitrocellulose membrane. Size selection was performed taking all material above 50 kDa (size of our free protein), and elution of RNAs was achieved by proteinase K (1X, Roche Applied Science) treatment, acid phenol–chloroform extraction, and ethanol precipitation. SMInput samples, after membrane selection, were 5′ and 3′ dephosphorylated and 3′ RNA linker ligated. SMInput and eCLIP samples were reverse transcribed (RT), and cDNAs were purified by Exo1 (New England Biolabs) treatment to remove unused RT primers and alkaline treatment to remove RNAs, followed by ethanol precipitation. A 3′ DNA ligation step was then performed with a barcoded linker. Ligation products were then purified with Agencourt AMPure XP beads (Beckman Coulter) modified with a cutoff set at 50-mer. Final quantities of the libraries were estimated using qPCR with P3 and P5 primers, and final PCR was performed according to those cycles. PCR products were size-selected (175–300 bp) by PAGE and eluted by diffusion. Samples were then precipitated and quantified prior to single-end sequencing on a NextSeq 500 sequencer (Illumina). Detailed protocol can be found in Hocq et al [26].

### Read preprocessing and data mapping

We performed demultiplexing of raw reads using a custom script that identifies sample 5′ end barcodes. After barcode removal, human datasets were mapped to the human genome (hg38, Ensembl 85, with processed transcripts and pseudo genes masked) using STAR (version 2.7.9a). *Drosophila* datasets were mapped to the *Drosophila* genome (BDGP6.22.96). We applied PCR duplicate removal on the demultiplexed data with umitools -dedup. Intersection to the genome of reference was performed with intersectBed module from Bedtools, after trimming the read to 1 nucleotide, corresponding to the 5′ end (the crosslinking position), against a homemade GTF composed only by one representative transcript per gene, selecting the isoform with the maximum number of exons, using the longest exonic size as a tiebreaker.

The number of reads at each step was calculated as follows: for “raw files”, the count was determined by counting fastq lines; for “.bam files”, before and after deduplication, bamtools - view was used; for reads mapping to coding sequences, the count was based on the number of lines of bed files after intersection.

### Meta-exon plot

For the distribution of the 5′ ends relative to the exon junction, bed files after GTF intersection were used. The distance of the 5′ end of each read was plotted to either the start or the end of the exon, depending on the strand, dividing the counts at each relative position by the number of exons covered at that position and the total number of mapped reads, to correct for exon length and library size, respectively.

### PureCLIP peak detection and Jaccard index

PureCLIP analysis was performed with the aligned files previously mentioned after deduplication. Merged bam SMinputs were used as a control to homogenize control populations over comparisons. The reference was a fasta file of the *Drosophila* genome BDGP6. A window of 10 nt around each PureCLIP binding region was added with bedtools -slopbed of 5 bp on both ends. To calculate peak agreement between replicates, bedtools -intersectBed was used. Then, the Jaccard index (J) metric was used to calculate the similarity between replicates. It is defined as:

$$J\left(A,B\right)=\frac{\left|A\cap B\right|}{\left|A\cup B\right|}=\frac{\left|A\cap B\right|}{\left|A\right|+\left|B\right|-\left|A\cap B\right|}$$

Being A the peaks from replicate 1 and B the peaks from replicate 2.

### ENCODE PureCLIP

We acquired a total of 84 RBP eCLIP datasets from the ENCODE project (https://www.encodeproject.org/). Our selection criteria included datasets that had a minimum of two replicates and at least one SMInput control in the repository. We detected peaks with PureCLIP as previously mentioned and computed the Jaccard indexes for each RBP.

### SRD development and meta-exons

The Stop Rate Difference (SRD) score was created from the JACUSA2 output [35]. We performed RT-arrest analysis independently on three replicates of *Drosophila* eIF4A3-eCLIP using a control dataset comprising the two unified SMInput sequencing results. We then identified positions where at least two replicates exhibited a significantly higher stop rate compared to the control, indicating specific EJC binding (*P* value < 0.05 on the integrated chi-square test). To obtain the SRD score, we took the mean of the stop rates per nucleotide of the three replicates and subtracted the stop rate of the control of the same nucleotide. Subsequently, to obtain the SRD score per exon, we calculated the average SRD score of all significant positions within a 10-bp window from − 23 to − 32 upstream of the exon junction of each exon.

Given that SRD score calculations involve treating the replicates collectively by restricting the analysis to positions significant in at least 2 out of the 3 replicates, it is not possible to compute the Jaccard index for the identified exons.

*Window at* − *18:* The analyses were performed in the same way as with the − 27 window. Then, we selected those peaks significant in 3 replicates in a window of 10 nucleotides around -18 and compared their exon_id with the peaks significant at − 27 in 3 replicates .

### IpaRt score development

To enable exon-level binding analysis for ipaRt, we used Bedtools to calculate genome coverage counts per position. Then, filtered out positions with a coverage of 0. We intersected the identified positions with the reference genome. For further analysis, we selected a specific window ranging from − 10 to − 36. Within this window, we computed the median coverage at each position. We kept a value per exon and calculated the average window median coverage for the 3 replicates. For mRNA sequencing data, we followed a similar process as the ipaRt replicates. We calculated the average of the median coverage for both mRNA sequencing windows. To evaluate the enrichment level, we computed the ratio by dividing the average of the median coverage in the ipaRt samples by the average of the median coverage in the mRNA sequencing samples. Finally, we focused on the exons that were consistently detected, meaning they had reads in all IPs and mRNA sequencing samples. We considered exons as loaded if the ratio in the window was superior to a threshold corresponding to the 95th percentile in the same window among the last exons.

### Other computational analyses

Quantifications, calculations, and plots for all subsequent analyses (unless clarified otherwise) were performed by Python v3.

### Supplementary Information

**Additional file 1.** **Figure S1.** Complexity for human and *Drosophila* eIF4A3 eCLIP libraries a) Bar plot displaying the read counts at successive steps of read analysis for each sample, including raw reads, uniquely mapped reads, deduplicated reads, and reads that intersect coding exons in the human data. Note that SMInput 2 appears as having 0 reads, due to very low output and plot scale b) The same as a) for *Drosophila* eCLIP data. c) Venn diagram depicting the similarity of eCLIP binding sites after PureCLIP binding site discovery for all *Drosophila* replicates. The Jaccard index (JI) on the right of each Venn diagram is calculated based on the values presented in the Venn diagram. **Figure S2.** Comparison of human and *Drosophila* transcriptomes and validation of CRISPR editing of *Drosophila* S2R+ cells a) Distribution of the number of exons per transcript in *D. melanogaster* and *H. sapiens*. b) Total number of annotated exons in *D. melanogaster* and *H. sapiens*. *Drosophila* annotations were obtained from the FlyBase consortium, version BDGP6. Human annotations were obtained from Ensembl, version GRCh38; only the longest isoform and with experimental evidence were used for quantification. c) Western blot to validate expression of endogenous eIF4A3 cells fused to 3xHA in S2R+. Anti-HA membrane (right) with lane 1 from a modified and single isolated clone, and lane 2 with wild-type cells. Anti-eIF4A3 *Drosophila* membrane (left) with lane 1 from a modified and single isolated clone and lane 2 with wild-type cells. The red arrow points to the expected size for endogenous eIF4A3, which becomes fainter in the S2 HA (lane 1). The blue arrow points to the expected size for eIF4A3-HA, which appears only upon CRISPR-cas9 and is absent in the wild type, with expression levels similar to eIF4A3-WT in WT cells. d) Co-IP of Y14-FLAG pulled down by eIF4A3-HA. Lane 1: input, with both proteins expressed. Lane 2: IP of eIF4A3 co-precipitates Y14-FLAG. **Figure S3.** eCLIP biases a) The scatter plot displays the Stop Rate scores per position for replicates 1 and 2 of *Drosophila* eIF4A3 eCLIP. The Spearman correlation score is shown on top. b) As in a) but for *Drosophila* eIF4A3 eCLIP datasets analyzed by PureCLIP. c) Boxplot showing the correlation between expression level and U count for EJC detection. Exons were divided in 10 different classes of equal bins and further divided into 7 classes depending on its U count in the EJC interacting window. SRD scores (y-axis) were plotted. **Figure S4.** ipaRt biasesa) IGV screenshot comparing eCLIP replicate 1 and ipaRt replicate 1, illustrating differences in both overall enrichment and background noise for the *Galk* gene. b) Stacked bar plot showing the count of detected exons versus total exons per exon group, including first exons, internal exons and last exons. c) Box plot comparing T counts in the window for detected and undetected exons in ipaRTs.

**Additional file 2.** **Table S1.** Peaks detected with PureCLIP in human eIF4A3 eCLIP replicates. **Table S2.** Comparison of peak detection between PureCLIP and Stop Rate Difference. **Table S3.** Distribution of detected peaks in SRD. **Table S4.** Distribution of detected peaks in ipaRt

**Additional file 3.** **Table S5.** Top 50 more expressed and reproducibly undetected exons (.txt)

## Data Availability

The datasets supporting the conclusions of this article are available in the SRA repository (accession number: PRJNA996108 [47]). ipaRt datasets were directly obtained from: [27]; https://www.ebi.ac.uk/ena/browser/view/PRJEB26421.
