# Supplementary material for: Comprehensive mapping of exon junction complex binding sites reveals universal EJC deposition in Drosophila
Source: BMC Biol. 2023 Nov 7;21:246. doi: 10.1186/s12915-023-01749-1 (PMC10630996; doi:10.1186/s12915-023-01749-1)
Supplement: Supplementary file 2 — Additional file 2. Table S1. Peaks detected with PureCLIP in human eIF4A3 eCLIP replicates. Table S2. Comparison of peak detection between PureCLIP and Stop Rate Difference. Table S3. Distribution of detected peaks in SRD. Table S4. Distribution of detected peaks in ipaRt [file 12915_2023_1749_MOESM2_ESM.pdf]

Table S1: Peaks detected with PureCLIP in human eIF4A3 eCLIP replicates
Columns indicate sample id and peaks detected, final column indicates the peaks that are common in both replicates

Sample	eCLIP 1	eCLIP 2	Common
Peaks	6947	5097	1815

Table S2: Comparison of peak detection between PureCLIP and Stop Rate Difference
Columns indicate the amount of peaks detected per each replicate, last column indicates those detected in common. In the case of PureCLIP comparisons were done two by two. In the case of SRD since we filter by significance and only consider those peaks significant in all replicates we only have the value of commonly detected peaks.

Sample	eCLIP 1	eCLIP 2	eCLIP 3	Common
PureCLIP peaks	26031	21738	16441	20401<br>15981<br>15996
SRD peaks				15246<br>(3 Replicates)

Table S3: Distribution of detected peaks in SRD.
Each column represents the amount of peaks detected corresponding to first, internal and last exons. First row corresponds to those that are detected as loaded, second row corresponds to the total distribution of exons in the drosophila transcriptome. Data extracted from mRNA-seq. Third row corresponds to the percentage of loaded compared to all expressed exons for each class.

	First	Internal	Last
Loaded (11295)	1928	9350	17
Total (23426)	3744	15832	3850
%	51%	59%	0.4%

Table S4: Distribution of detected peaks in ipaRt.
Each column represents the amount of peaks detected corresponding to first, internal and last exons. First row corresponds to those that are detected as loaded, second row corresponds to the total distribution of exons in the drosophila transcriptome. Data extracted from mRNA-seq. Third row corresponds to the percentage of loaded compared to all expressed exons for each class.

	First	Internal	Last
Loaded (30200)	5925	23700	543
Total (49700)	8800	32200	8600
%	67%	73%	6%
